# A combined association of alanine aminotransferase, aspartate transaminase and bilirubin with sleep duration in aged 16–85 years (2005–2010)

**DOI:** 10.1097/MD.0000000000040915

**Published:** 2024-12-06

**Authors:** Lishuai Yao, Tiantian Chen

**Affiliations:** a Department of Cardiothoracic Surgery, The Third Affiliated Hospital of Sun Yat-sen University, Sun Yat-sen University, Guangzhou, China.

**Keywords:** alanine aminotransferase, aspartate transaminase, bilirubin, National Health and Nutrition Examination Survey, nonalcoholic fatty liver disease, sleep duration

## Abstract

Sleep is a vital restorative process that plays a pivotal role in maintaining the delicate equilibrium of mental and physical well-being. Both short and long sleep duration are associated with a range of adverse health outcomes. Numerous studies have consistently demonstrated a robust association between sleep duration and liver disease. In this study, we conducted statistical tests and performed subgroup analyses to explore potential variations in this association across different contexts, aiming to elucidate the correlation between ALT, AST, and TB with sleep duration. This cross-sectional investigation utilized datasets from the National Health and Nutrition Examination Survey 2005 to 2010. Multivariate linear regression models were used to examine the linear association between ALT, AST, and TB with sleep duration. Test for interaction is commonly conducted using multivariabte models to assess statistically significant subgroup disparities. Fitted smoothied curves and threshold effect analyses were employed to depict nonlinear relationships. The study enrolled 17,491 participants aged 16 to 85 years who met the inclusion and exclusion criteria, with a mean age of the participants was 45.58 ± 19.94 years. Multivariate linear regression analysis showed a significant positive association between sleep duration and ALT [−0.23 (−0.45, −0.00) 0.0455] and AST[−0.20 (−0.38, −0.01) 0.0338] in Model 3. Using a two-segment linear regression model, we found an U-shaped relationship and significant inflection point between between ALT and AST with sleep duration. The present study unveiled a significant inverse correlation between sleep duration and levels of ALT and AST, while no significant association was observed with TB levels. Furthermore, variations in the optimal sleep duration for liver function recovery were identified across diverse populations, thereby offering valuable healthcare recommendations to public.

## 1. Introduction

Sleep is a vital restorative process that plays a pivotal role in maintaining the delicate equilibrium of mental and physical well-being. Sleep serves as an essential mechanism for optimizing the functionality of every organ within the human body, and its deprivation can precipitate pathological conditions and irreversible harm.^[1]^

Both short and long sleep durations are associated with a range of adverse health outcomes. Significant associations have been observed between short sleep duration and outcomes such as mortality,^[2]^ diabetes,^[3]^ hypertension,^[4]^ cardiovascular disease,^[5]^ and coronary heart disease.^[6]^ The causal association between short sleep duration and adverse health outcomes is supported by meta-analyses of epidemiological and cohort data^[7,8]^ as well as laboratory studies on experimental sleep restriction and deprivation.^[9,10]^ Systematic evaluations have demonstrated that prolonged sleep is linked to important health outcomes, including not only mortality,^[11–14]^ but also cardiovascular disease^[15]^ stroke,^[16,17]^ and diabetes.^[18,19]^

Numerous studies have consistently demonstrated a robust association between sleep duration and liver disease. Insufficient sleep duration and sleep disorders have been linked to abnormal serum aminotransferase activity and nonalcoholic fatty liver,^[20–24]^ as well as an increased risk of cirrhosis in patients with NAFLD.^[25]^ A study conducted on participants from the UK Biobank, encompassing cases of viral hepatitis, NAFLD, cirrhosis, alcoholic liver disease, and liver cancer, revealed that maintaining healthy sleep patterns was inversely associated with the development of chronic liver disease. Moreover, individuals at higher genetic susceptibility were found to be more prone to developing chronic liver disease when exposed to unhealthy sleep patterns.^[26]^

Alanine aminotransferase (ALT), aspartate aminotransferase (AST), and total bilirubin (TB) serve as crucial indicators of hepatic function. However, to the best of our knowledge, there is currently a dearth of comprehensive investigations exploring the relationship between these biomarkers and sleep duration. In this study, we conducted statistical tests and performed subgroup analyses to explore potential variations in this association across different contexts, aiming to elucidate the correlation between ALT, AST, and TB with sleep duration.

## 2. Methods

### 2.1. Study population

The National Health and Nutrition Examination Survey (NHANES) is a cross-sectional study conducted by the National Center for Health Statistics of the U.S. Centers for Disease Control and Prevention. It employs a rigorous, complex, multistage, probability sampling methodology to provide comprehensive insights into the nutritional and health status of the entire U.S. population.^[27]^

**Figure 1.**
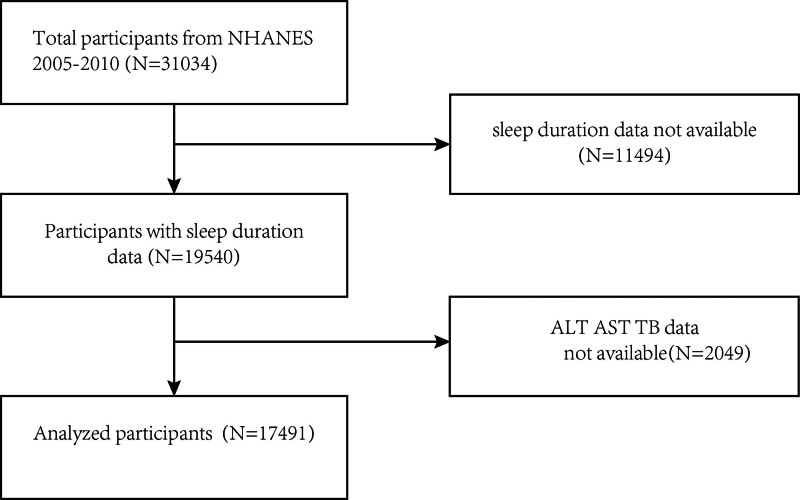
Sample selection flowchart.

This survey used consecutive cycles of the 2005 to 2010 NHANES data, which included 31,034 participants. We excluded 19,540 participants with missing sleep duration data and 2049 participants with missing ALT, AST, and TB data. The study eventually included 17,491 participants. Figure 1 illustrates the sample selection flowchart.

### 2.2. Study variables

The study’s dependent variable was sleep duration, while ALT, AST, and TB were proposed as independent variables. The DxC800 analyzer utilizes a kinetic rate method for the quantification of ALT levels in serum or plasma, while employing an enzymatic rate method to measure AST levels. The concentration of TB is determined in serum or plasma using the timed endpoint diazo method (Jendrassik-Grof) on the same analyzer. The covariates considered in this study included age, gender, race, income-to-poverty ratio, education level, moderate activities status, smoking status (if smoked at least 100 cigarettes in life), body mass index (BMI), waist circumference, ALB (albumin), creatinine (CR), and uric acid (UA).

### 2.3. Statistical analysis

Statistical analyses were conducted using the statistical computing and graphics software R (version 4.2.0) and EmpowerStats (www.empowerstats.com, version: 4.2). The baseline characteristics of the study population were described based on the tertile of sleep duration, respectively short sleep duration (1–5 hours), middle sleep duration (6–7 hours) and long sleep duration (8–12 hours). Continuous variables are presented as mean (standard deviation (SD)), and categorical variables are summarized as frequency (percentage (%)). Considering the complexity of the sampling method, the study subject was a weighted statistical analysis (Weighted by: WTMECPRP). Beta values and their corresponding 95% confidence intervals were estimated using multiple linear regression analyses of ALT, AST, and TB with sleep duration. Multivariate tests were performed with 3 models: Model 1 included no variable adjustment; Model 2 was adjusted for age and ethnicity; and Model 3 was adjusted for all covariates. We conducted subgroup analysis to evaluate the heterogeneity between ALT, AST, and TB with sleep duration based on the following variables: gender, age, race, education levels, moderate activities status, smoking status (if smoked at least 100 cigarettes in life) and BMI. Smoothed curve fits were simultaneously conducted by adjusting for relevant variables to explore the relationship between ALT, AST, and TB with sleep duration as well as identifying potential inflection points using a threshold effects analysis model. Finally, the same statistical study methods were used for performed for subgroups, and an interaction test was performed. It was determined that *P* < .05 was statistically significant. We use a weighting method to reduce the significant volatility of our dataset.

## 3. Results

### 3.1. Baseline characteristics

The study enrolled 17,491 participants aged 16 to 85 years who met the inclusion and exclusion criteria, with a mean age of the participants was 45.58 ± 19.94 years.

Among these individuals, 49.06% were men, 50.94% were women, Mexican Americans for 20.0%, other Hispanics for 8.52%, non-Hispanic whites for 46.38%, non-Hispanic blacks for 20.43%, and other races for 4.67% of the sample population. The average (SD) ALT, AST, and TB levels were 25.27 (19.96) IU/L, 25.99 (16.27) IU/L, and 12.80 (5.48) μmol/L, respectively.

Table 1 lists weighted characteristics of the study population based on the tertile of sleep duration. According to sleep duration, ALT, AST, and TB levels showed significant differences in the different tertiles (*P* < .001). The long sleep duration group exhibited decreased levels of ALT and AST compared to the short sleep duration group, while displaying elevated levels of TB. Furthermore, sleep duration were significantly associated with gender, race, education level, income-to-poverty ratio, BMI, waist circumference, ALB, UA, activities status and smoking status (*P* < .05). Nonetheless, sleep duration were not significantly correlated with age and CR (*P* > .05).

**Table 1:** Weighted characteristics of the study population based on the tertile of sleep duration

Sleep duration	Short (1–5 h) N = 2609	Middle (6–7 h) N = 8562	Long (8–12 h) N = 6320	*P* value
AGE (years)	45.55 ± 16.70	44.71 ± 16.75	44.92 ± 19.55	.1297
GENDER (%)				<.0001
Men	49.80	50.35	45.01	
Women	50.20	49.65	54.99	
Ethnicity (%)				<.0001
Mexican American	7.38	8.15	9.91	
Other Hispanic	5.66	4.61	4.12	
Non-Hispanic White	58.67	71.31	71.37	
Non-Hispanic Black	20.91	9.74	9.09	
Other race	7.38	6.19	5.52	
Education level (%)				<.0001
Less than high school	25.07	16.32	20.63	
High school	27.12	23.97	23.32	
More than high school	47.81	59.72	56.05	
PIR (%)	2.56 ± 1.61	3.18 ± 1.61	2.95 ± 1.63	<.0001
BMI (kg/m^2^)	29.57 ± 7.42	28.40 ± 6.37	27.83 ± 6.78	<.0001
Waist circumference (cm)	99.71 ± 16.97	97.19 ± 15.95	95.73 ± 16.49	<.0001
ALB (g/L)	42.14 ± 3.52	42.77 ± 3.35	42.58 ± 3.59	<.0001
ALT (IU/L)	26.59 ± 16.86	25.90 ± 19.82	24.67 ± 15.79	<.0001
AST (IU/L)	26.67 ± 18.39	25.68 ± 14.11	25.47 ± 11.66	.0019
TB (μmol/L)	12.63 ± 4.97	13.19 ± 5.44	12.94 ± 5.30	<.0001
CR (μmol/L)	79.74 ± 35.91	78.57 ± 25.99	78.63 ± 31.34	.2167
UA (μmol/L)	326.67 ± 85.44	324.73 ± 83.44	317.74 ± 83.45	<.0001
Moderate activities (%)				<.0001
Yes	45.80	49.12	43.29	
No	54.20	50.88	56.71	
Smoked at least 100 cigarettes				<.0001
Yes	54.67	45.79	45.50	
No	45.33	54.21	54.50	

Mean ± SD for: AGE, PIR, BMI, WAIST, ALB, ALT, AST, TB, CR, UA. *P* value was calculated by weighted linear regression model.

% for: GENDER, Ethnicity Education level, Moderate activities, Smoked at least 100 cigarettes. *P* value was calculated by weighted chi-square test.

Weighted by: WTMEC2YR.

ALB = albumin, ALT = alanine aminotransferase, AST = aspartate transaminase, BMI = body mass index, CR = creatinine, PIR = income-to-poverty ratio, TB = total bilirubin, UA = uric acid.

### 3.2. Association between sleep duration and ALT

Table 2 shows the results of the multivariate regression analysis. In the unadjusted model, sleep duration were highly negative correlated with ALT level [−0.53 (−0.73, −0.34) < .0001]. This negative correlation remained significant in Model 2 [−0.46 (−0.66, −0.27) < .0001] and Model 3[−0.23 (−0.45, −0.00) .0455]. In subgroup analyses stratified by age or gender, our findings demonstrated a significant negative correlation in age ≤ 40 years group [−0.64 (−0.94, −0.34) < .0001] and men [−0.75 (−1.08, −0.43) < .0001] in Model 2. In subgroup analyses stratified by BMI or smoking status, sleep duration showed a significant negative correlation with ALT level in low-BMI group [−0.34 (−0.58, −0.11) .0046] and nonsmoking group [−0.48 (−0.75, −0.21) .0006], while those significant negative correlation became insignificant in Model 3.

**Table 2:** The association between sleep duration and ALT

	Model 1 β (95% CI) *P* value	Model 2 β (95% CI) *P* value	Model 3 β (95% CI) *P* value
ALT (IU/L)	−0.53 (−0.73, −0.34) < .0001	−0.46 (−0.66, −0.27) < .0001	−0.23 (−0.45, −0.00) .0455
Stratified by age			
AGE ≤ 40	−0.70 (−1.01, −0.39) < .0001	−0.64 (−0.94, −0.34) < .0001	−0.19 (−0.56, 0.18) .3105
AGE > 40	−0.40 (−0.65, −0.15) .0018	−0.30 (−0.55, −0.06) .0163	−0.24 (−0.52, 0.03) .0849
Stratified by gender			
Men	−0.65 (−0.97, −0.32) < .0001	−0.75 (−1.08, −0.43) < .0001	−0.34 (−0.71, 0.04) .0808
Women	−0.18 (−0.40, 0.03) .0946	−0.22 (−0.43, −0.00) .0471	−0.08 (−0.33, 0.17) .5141
Stratified by the tertile of BMI			
Low	−0.46 (−0.70, −0.22) .0002	−0.34 (−0.58, −0.11) .0046	−0.26 (−0.54, 0.03) .0818
Middle	−0.40 (−0.76, −0.03) .0321	−0.34 (−0.69, 0.01) .0598	−0.26 (−0.66, 0.14) .1997
High	−0.13 (−0.53, 0.27) .5202	−0.13 (−0.51, 0.25) .5017	−0.07 (−0.50, 0.35) .7398
Smoked at least 100 cigarettes			
Yes	−0.35 (−0.67, −0.03) .0310	−0.25 (−0.56, 0.07) .1255	−0.18 (−0.52, 0.15) .2908
No	−0.58 (−0.86, −0.30) < .0001	−0.48 (−0.75, −0.21) .0006	−0.26 (−0.55, 0.03) .0813

Model I: no covariates were adjusted. Model II: AGE, GENDER and Ethnicity were adjusted. Model III: AGE; GENDER; Ethnicity; PIR; Moderate activities; Smoked at least 100 cigarettes; BMI level; WAIST circumference; ALB; CR; Education level; UA were adjusted. In the subgroup analysis stratified by (age/gender/BMI/smoking status), the model is not adjusted for (age/gender/BMI/smoking status). Weighted by: WTMEC2YR.

ALB = albumin, ALT = alanine aminotransferase, BMI = body mass index, CR = creatinine, PIR = income-to-poverty ratio, UA = uric acid.

In the association between the quartile of sleep duration and ALT, it revealed a statistically significant negative *P* value for trend in all Model (*P* for trend = .0195 in Model 3) (Table 3).

**Table 3:** The association between the tertile of sleep duration and ALT

	Model 1 β (95% CI) *P* value	Model 2 β(95% CI) *P* value	Model 3 β (95% CI) *P* value
Tertile of sleep duration			
Low (1–5 h)	Reference	Reference	Reference
Middle (6–7 h)	−0.69 (−1.52, 0.14) .1028	−0.97 (−1.77, −0.16) .0189	−0.93 (−1.85, −0.02) .0460
High (8–12 h)	−1.92 (−2.79, −1.05) < .0001	−1.83 (−2.68, −0.98) < .0001	−1.17 (−2.14, −0.20) .0178
*P* for trend	−0.67 (−0.96, −0.38) < .0001	−0.62 (−0.90, −0.34) < .0001	−0.38 (−0.71, −0.06) .0195

Model I: no covariates were adjusted. Model II:AGE, GENDER and Ethnicity were adjusted. Model III: AGE; GENDER; Ethnicity; PIR; Moderate activities; Smoked at least 100 cigarettes; BMI level; WAIST circumference; ALB; CR; Education level; UA were adjusted. Weighted by: WTMEC2YR.

ALB = albumin, ALT = alanine aminotransferase, BMI = body mass index, CR = creatinine, UA = uric acid.

Subgroup analyses were conducted to examine the association between sleep duration tertiles and ALT levels (Table 4). Subgroup analysis was performed based on gender, age, race, education levels, moderate activity status, smoking status and BMI. Statistical significance was found in gender subgroups (*P* for interaction = .0226) of ALT level. Subgroup analysis showed that ALT level of non-Hispanic White group was more sensitive to sleep duration compared with another subgroup (*P* for trend = .0140).

**Table 4:** Subgroup analyses of the association between the tertile of sleep duration and ALT

Sleep duration	Low (1–5 h)	Middle (6–7 h)	High (8–12 h)	*P* for trend	*P* for interaction
Gender					.0226
Men	Reference	−0.78 (−2.28, 0.71) .3058	−0.98 (−2.59, 0.63) .2336	−0.32 (−0.86, 0.21) .2380	
Women	Reference	−0.80 (−1.88, 0.27) .1433	−1.00 (−2.12, 0.12) .0800	−0.32 (−0.70, 0.05) .0858	
Age (years)					.6680
≤40	Reference	−1.37 (−2.90, 0.16) .0787	−1.23 (−2.85, 0.39) .1374	−0.38 (−0.92, 0.15) .1615	
>40	Reference	−0.60 (−1.74, 0.54) .3012	−1.08 (−2.29, 0.12) .0789	−0.37 (−0.77, 0.03) .0736	
Ethnicity (%)					.7400
Mexican American	Reference	1.71 (−1.98, 5.40) .3643	0.55 (−3.27, 4.38) .7763	0.08 (−1.18, 1.34) .9060	
Other Hispanic	Reference	−4.89 (−8.07, −1.72) .0026	−2.03 (−5.49, 1.44) .2514	−0.75 (−1.91, 0.41) .2038	
Non-Hispanic White	Reference	−1.37 (−2.64, −0.09) .0352	−1.71 (−3.04, −0.38) .0119	−0.55 (−0.99, −0.11) .0140	
Non-Hispanic Black	Reference	−0.45 (−1.93, 1.04) .5532	−0.28 (−1.97, 1.40) .7400	−0.12 (−0.67, 0.42) .6624	
Other Race	Reference	0.23 (−3.17, 3.63) .8950	−0.12 (−3.83, 3.59) .9481	−0.04 (−1.27, 1.20) .9543	
Moderate activities (%)					.8671
Yes	Reference	−0.80 (−2.11, 0.51) .2313	−1.01 (−2.41, 0.40) .1592	−0.33 (−0.80, 0.14) .1647	
No	Reference	−1.08 (−2.36, 0.21) .1002	−1.35 (−2.69, −0.01) .0480	−0.44 (−0.89, 0.00) .0514	
Smoked at least 100 cigarettes					.8782
Yes	Reference	−0.33 (−1.72, 1.06) .6422	−0.99 (−2.47, 0.50) .1927	−0.33 (−0.83, 0.16) .1877	
No	Reference	−1.52 (−2.73, −0.31) .0137	−1.39 (−2.66, −0.12) .0322	−0.41 (−0.83, 0.01) .0529	
Education level (%)					.9279
Less than high school	Reference	−0.47 (−2.83, 1.89) .6971	−0.87 (−3.32, 1.58) .4863	−0.29 (−1.11, 0.53) .4859	
High school	Reference	−1.86 (−3.53, −0.18) .0304	−1.82 (−3.61, −0.03) .0469	−0.60 (−1.19, −0.00) .0499	
More than high school	Reference	−0.69 (−1.83, 0.46) .2400	−1.01 (−2.23, 0.20) .1014	−0.34 (−0.74, 0.06) .0986	
The tertile of BMI					.8041
Low	Reference	−0.71 (−2.00, 0.57) .2745	−1.14 (−2.47, 0.19) .0928	−0.38 (−0.82, 0.05) .0841	
Middle	Reference	−1.46 (−3.09, 0.17) .0798	−0.77 (−2.49, 0.95) .3793	−0.21 (−0.78, 0.36) .4791	
High	Reference	−0.64 (−2.33, 1.05) .4565	−1.31 (−3.14, 0.51) .1584	−0.44 (−1.05, 0.17) .1595	

AGE; GENDER; Ethnicity; PIR; Moderate activities; Smoked at least 100 cigarettes; BMI level; WAIST circumference; ALB; CR; Education level; UA were adjusted. In the subgroup analysis stratified by (AGE, Ethnicity, etc.), the model is not adjusted for (AGE, Ethnicity, etc.). Weighted by: WTMEC2YR.

ALB = albumin, ALT = alanine aminotransferase, BMI = body mass index, CR = creatinine, UA = uric acid.

**Figure 2.**
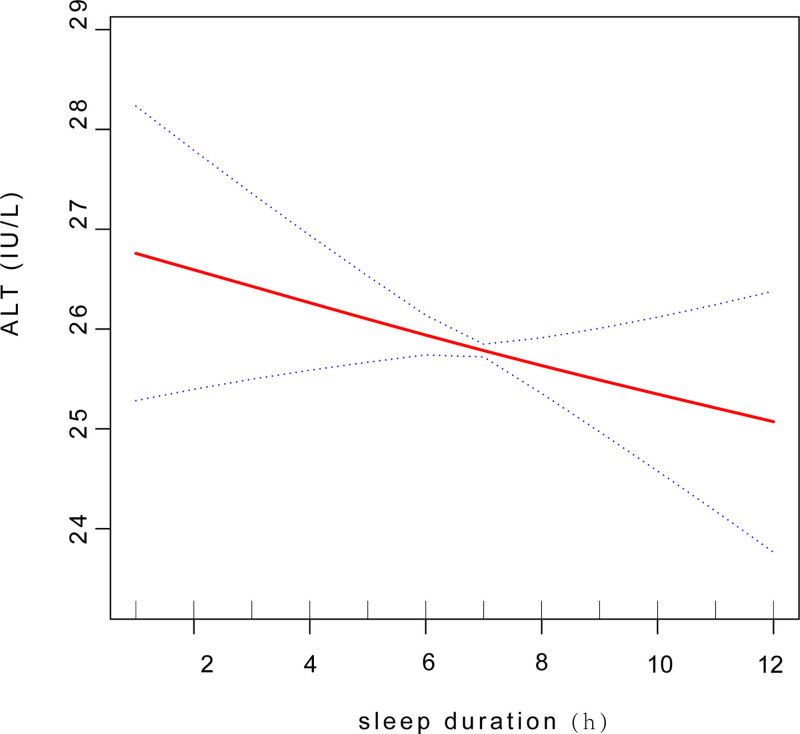
Smooth curve fit between sleep duration and ALT level. ALT = alanine aminotransferase.

**Figure 3.**
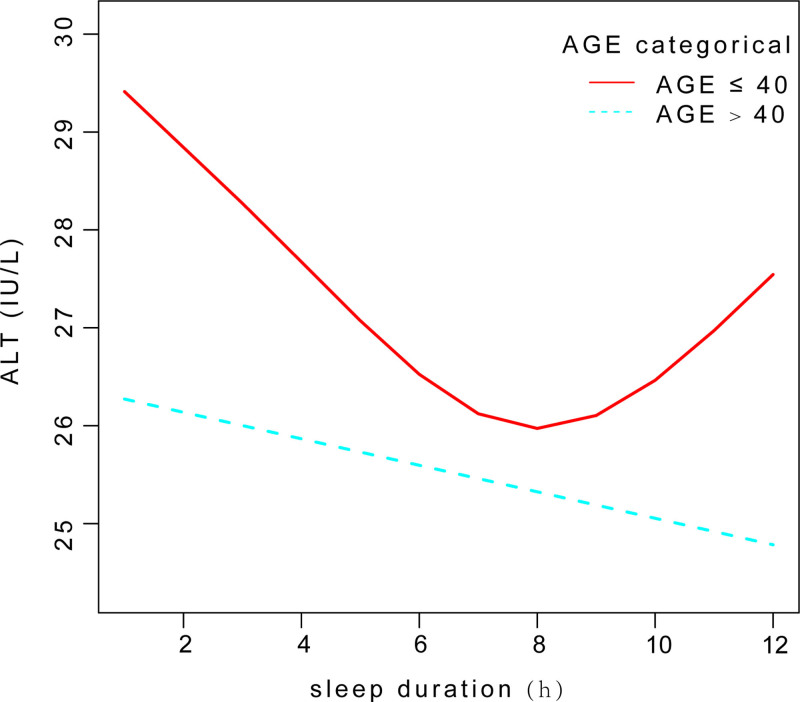
Smooth curve fit between sleep duration and ALT level in subgroup analyses stratified by age. ALT = alanine aminotransferase.

We employed a smooth curve fit to accurately depict the non-linear association between sleep duration and ALT level (Fig. 2). Using a two-segment linear regression model, in subgroup analyses stratified by age, we found an U-shaped relationship between sleep duration and ALT level with an inflection point of 8 hours and a significant log-likelihood ratio (*P *= .019) in age ≤ 40 years group (Fig. 3). A negative correlation was observed when sleep duration < 8 hours [−0.33 (−0.78, 0.12) .1509]. Conversely, when sleep duration > 8 hours, a significant positive correlation was found between sleep duration and ALT levels[1.41 (0.13, 2.69) .0306](Table 5).

**Table 5:** Threshold effect analysis of sleep duration on ALT using two-piecewise linear regression model

ALT(IU/L)	Adjusted β (95% CI) *P* value
Sleep duration	
Inflection point	8
<8 h	−0.37 (−0.65, −0.08) .0117
>8 h	0.56 (−0.26, 1.37) .1787
Log likelihood ratio	.050
Stratified by age	
≤40 years	
Inflection point	8
<8 h	−0.33 (−0.78, 0.12) .1509
>8 h	1.41 (0.13, 2.69) .0306
Log likelihood ratio	.019
>40 years	
Inflection point	4
<4 h	−2.15 (−4.91, 0.60) .1248
>4 h	−0.21 (−0.55, 0.12) .2087
Log likelihood ratio	.180
The tertile of BMI	
Low	
Inflection point	8
<8 h	−0.20 (−0.55, 0.16) .2794
>8 h	−0.47 (−1.30, 0.35) .2621
Log likelihood ratio	.580
Middle	
Inflection point	5
<5 h	−3.36 (−5.11, −1.61) .0002
>5 h	0.18 (−0.28, 0.64) .4483
Log likelihood ratio	<.001
High	
Inflection point	8
<8 h	−0.44 (−0.94, 0.06) .0846
>8 h	1.97 (0.48, 3.47) .0098
Log likelihood ratio	.005

AGE; GENDER; Ethnicity; PIR; Moderate activities; Smoked at least 100 cigarettes; BMI level; WAIST circumference; ALB; CR; Education level; UA were adjusted. In the subgroup analysis stratified by (age/BMI), the model is not adjusted for (age/BMI). Weighted by: WTMEC2YR.

ALB = albumin, ALT = alanine aminotransferase, BMI = body mass index, CR = creatinine, UA = uric acid.

**Figure 4.**
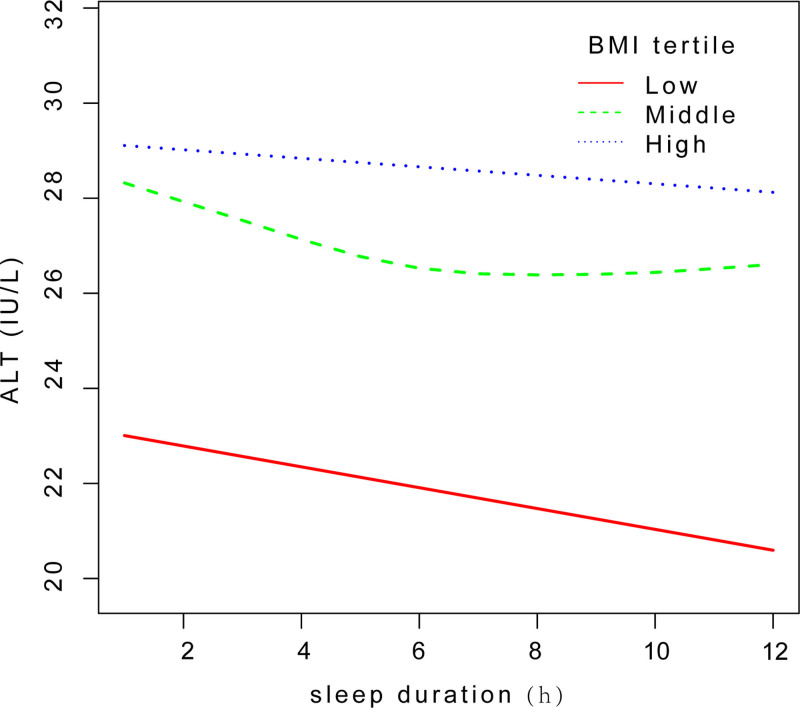
Smooth curve fit between sleep duration and ALT level in subgroup analyses stratified by BMI. ALT = alanine aminotransferase, BMI = body mass index.

In subgroup analyses stratified by the tertile of BMI, it showed an U-shaped relationship between sleep duration and ALT level in middle-BMI group with an inflection point of 5 hours (log-likelihood ratio* < *0.001) and high-BMI group with an inflection point of 8 hours (log-likelihood ratio* *= 0.005) (Table 5; Fig. 4).

### 3.3. Association between sleep duration and AST

Table 6 shows the results of the multivariate regression analysis. In the unadjusted model, sleep duration were highly negative correlated with AST level [−0.28 (−0.43, −0.13) .0003]. This negative correlation remained significant in Model 2 [−0.20 (−0.35, −0.05) .0093] and Model 3 [−0.20 (−0.38, −0.01) .0338].

**Table 6:** The association between sleep duration and AST

	Model 1 β (95% CI) *P* value	Model 2 β (95% CI) *P* value	Model 3 β (95% CI) *P* value
AST(IU/L)	−0.28 (−0.43, −0.13) .0003	−0.20 (−0.35, −0.05) .0093	−0.20 (−0.38, −0.01) .0338
Stratified by age			
AGE ≤ 40	−0.37 (−0.62, −0.12) .0032	−0.30 (−0.54, −0.06) .0162	−0.27 (−0.60, 0.06) .1031
AGE > 40	−0.18 (−0.37, 0.01) .0646	−0.10 (−0.29, 0.09) .3028	−0.11 (−0.32, 0.10) .3089
Stratified by gender			
Men	−0.38 (−0.61, −0.14) .0016	−0.34 (−0.58, −0.10) .0047	−0.28 (−0.56, 0.01) .0569
Women	−0.08 (−0.27, 0.11) .4072	−0.08 (−0.27, 0.11) .4302	−0.09 (−0.32, 0.13) .4230
Stratified by the tertile of BMI			
Low	−0.27 (−0.48, −0.07) .0100	−0.15 (−0.36, 0.05) .1441	−0.15 (−0.41, 0.11) .2481
Middle	−0.28 (−0.62, 0.05) .0980	−0.23 (−0.56, 0.11) .1866	−0.29 (−0.68, 0.10) .1411
High	−0.14 (−0.38, 0.10) .2597	−0.12 (−0.36, 0.12) .3454	−0.13 (−0.40, 0.15) .3626
Smoked at least 100 cigarettes			
Yes	−0.16 (−0.40, 0.07) .1756	−0.13 (−0.36, 0.10) .2783	−0.16 (−0.41, 0.09) .2132
No	−0.34 (−0.58, −0.11) .0041	−0.26 (−0.50, −0.03) .0280	−0.23 (−0.49, 0.03) .0781

Model I: no covariates were adjusted. Model II: AGE, GENDER and Ethnicity were adjusted. Model III: AGE; GENDER; Ethnicity; PIR; Moderate activities; Smoked at least 100 cigarettes; BMI level; WAIST circumference; ALB; CR; Education level; UA were adjusted. In the subgroup analysis stratified by (age/gender/BMI/smoking status), the model is not adjusted for (age/gender/BMI/smoking status). Weighted by: WTMEC2YR.

ALB = albumin, AST = aspartate transaminase, BMI = body mass index, CR = creatinine, UA = uric acid.

In subgroup analyses stratified by age or gender, our findings demonstrated a significant negative correlation in age ≤ 40 years group [−0.30 (−0.54, −0.06) .0162] and men [−0.34 (−0.58, −0.10) .0047] in Model 2. In subgroup analyses stratified by smoking status, sleep duration showed a significant negative correlation with AST level in nonsmoking group [−0.26 (−0.50, −0.03) .0280], while those significant negative correlation became insignificant in Model 3.

In the association between the quartile of sleep duration and AST, it revealed a statistically significant negative *P* value for trend in all Model (*P* for trend = .0317 in Model 3) (Table 7).

**Table 7:** The association between the tertile of sleep duration and AST

	Model 1 β (95% CI) *P* value	Model 2 β (95% CI) *P* value	Model 3 β (95% CI) *P* value
Tertile of sleep duration			
Low (1–5 h)	Reference	Reference	Reference
Middle (6–7 h)	−0.99 (−1.63, −0.35) .0024	−0.89 (−1.53, −0.26) .0060	−1.02 (−1.76, −0.27) .0075
High (8–12 h)	−1.19 (−1.87, −0.52) .0005	−0.92 (−1.59, −0.25) .0072	−0.92 (−1.71, −0.13) .0228
*P* for trend	−0.39 (−0.61, −0.17) .0006	−0.29 (−0.51, −0.07) .0100	−0.29 (−0.55, −0.03) .0317

Model I: no covariates were adjusted. Model II: AGE, GENDER and Ethnicity were adjusted. Model III: AGE; GENDER; Ethnicity; PIR; Moderate activities; Smoked at least 100 cigarettes; BMI level; WAIST circumference; ALB; CR; Education level; UA were adjusted. Weighted by: WTMEC2YR.

ALB = albumin, AST = aspartate transaminase, BMI = body mass index, CR = creatinine, UA = uric acid.

We also performed subgroup analyses of the association between the tertile of sleep duration and AST level (Table 8). Subgroup analysis was performed based on gender, age, race, education levels, moderate activity status, smoking status and BMI. The subgroups of ALT level did not exhibit statistical significance. Subgroup analysis showed that ALT level of non-Hispanic Black group, moderate activities group, nonsmoking group, and High school group was more sensitive to sleep duration compared with another subgroup(*P* for trend < .05).

**Table 8:** Subgroup analyses of the association between the tertile of sleep duration and AST

Sleep duration	Low (1–5 h)	Middle (6–7 h)	High (8–12 h)	*P* for trend	*P* for interaction
Gender					.0530
Men	Reference	−1.44 (−2.57, −0.30) .0131	−0.97 (−2.19, 0.25) .1183	−0.30 (−0.71, 0.10) .1436	
Women	Reference	−0.51 (−1.49, 0.48) .3120	−0.73 (−1.76, 0.30) .1631	−0.24 (−0.58, 0.10) .1625	
Age					.2374
≤ 40	Reference	−1.36 (−2.72, −0.01) .0486	−1.27 (−2.71, 0.16) .0827	−0.40 (−0.88, 0.08) .0998	
>40	Reference	−0.80 (−1.67, 0.07) .0724	−0.63 (−1.55, 0.29) .1814	−0.19 (−0.50, 0.11) .2181	
Ethnicity(%)					.5713
Mexican American	Reference	0.46 (−2.88, 3.80) .7872	−0.40 (−3.86, 3.05) .8196	−0.19 (−1.33, 0.95) .7396	
Other Hispanic	Reference	−2.50 (−4.51, −0.49) .0151	−1.12 (−3.31, 1.08) .3181	−0.41 (−1.14, 0.32) .2727	
Non-Hispanic White	Reference	−0.78 (−1.68, 0.11) .0863	−0.69 (−1.62, 0.25) .1503	−0.20 (−0.51, 0.11) .2028	
Non-Hispanic Black	Reference	−2.22 (−4.18, −0.27) .0261	−2.11 (−4.33, 0.10) .0620	−0.79 (−1.51, −0.07) .0319	
Other Race	Reference	−1.58 (−3.85, 0.70) .1759	−1.47 (−3.96, 1.01) .2456	−0.50 (−1.33, 0.33) .2364	
Moderate activities(%)					.2654
Yes	Reference	−0.74 (−1.59, 0.12) .0906	−0.63 (−1.54, 0.29) .1799	−0.20 (−0.50, 0.11) .2083	
No	Reference	−1.41 (−2.58, −0.23) .0188	−1.34 (−2.56, −0.11) .0325	−0.42 (−0.83, −0.01) .0428	
Smoked at least 100 cigarettes					.8160
Yes	Reference	−0.72 (−1.76, 0.32) .1764	−0.69 (−1.81, 0.42) .2228	−0.23 (−0.60, 0.14) .2284	
No	Reference	−1.47 (−2.56, −0.39) .0075	−1.33 (−2.47, −0.19) .0217	−0.40 (−0.77, −0.02) .0387	
Education level (%)					.3860
Less than high school	Reference	−0.17 (−2.01, 1.67) .8530	−1.04 (−2.96, 0.87) .2847	−0.35 (−0.99, 0.29) .2831	
High school	Reference	−2.16 (−3.53, −0.79) .0020	−1.53 (−2.98, −0.07) .0400	−0.49 (−0.98, −0.01) .0461	
More than high school	Reference	−0.86 (−1.83, 0.12) .0845	−0.73 (−1.77, 0.30) .1657	−0.21 (−0.56, 0.13) .2199	
The tertile of BMI					.6313
Low	Reference	−0.91 (−2.06, 0.23) .1178	−0.88 (−2.07, 0.31) .1466	−0.26 (−0.65, 0.13) .1894	
Middle	Reference	−1.69 (−3.28, −0.10) .0372	−0.90 (−2.57, 0.78) .2942	−0.24 (−0.80, 0.32) .3980	
High	Reference	−0.60 (−1.68, 0.48) .2747	−1.14 (−2.31, 0.02) .0540	−0.38 (−0.77, 0.01) .0545	

AGE; GENDER; Ethnicity; PIR; Moderate activities; Smoked at least 100 cigarettes; BMI level; WAIST circumference; ALB; CR; Education level; UA were adjusted. In the subgroup analysis stratified by (AGE, Ethnicity, etc.), the model is not adjusted for (AGE, Ethnicity, etc.). Weighted by: WTMEC2YR.

ALB = albumin, AST = aspartate transaminase, BMI = body mass index, CR = creatinine, PIR = income-to-poverty ratio, UA = uric acid.

**Figure 5.**
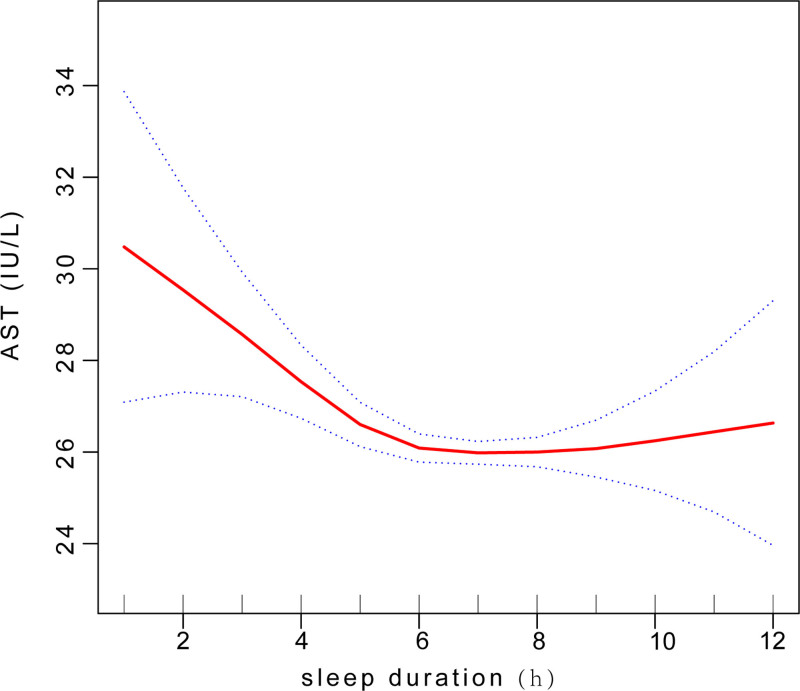
Smooth curve fit between sleep duration and AST level. AST = aspartate transaminase.

The non-linear relationship between sleep duration and AST level was accurately depicted by employing a smooth curve fit, revealing a U-shaped association (Fig. 5). The results revealed a significant negative correlation between sleep duration and AST levels when the sleep duration was < 5 hours [−1.80 (−2.65, −0.95) < .0001]. Conversely, a positive correlation was observed when the sleep duration > 5 hours [0.07 (−0.16, 0.29) .5592](Table 9).

**Table 9:** Threshold effect analysis of sleep duration on AST using two-piecewise linear regression model

AST (IU/L)	Adjusted β (95% CI) P value
Sleep duration	
Inflection point	5
<5 h	−1.80 (−2.65, −0.95) < .0001
>5 h	0.07 (−0.16, 0.29) .5592
Log likelihood ratio	<.001
Stratified by gender	
Men	
Inflection point	5
<5 h	−3.09 (−4.48, −1.70) < .0001
>5 h	0.09 (−0.28, 0.45) .6379
Log likelihood ratio	<.001
Women	
Inflection point	5
<5 h	−0.56 (−1.52, 0.39) .2490
>5 h	0.09 (−0.16, 0.34) .4795
Log likelihood ratio	0.227

AGE; GENDER; Ethnicity; PIR; Moderate activities; Smoked at least 100 cigarettes; BMI level; WAIST circumference; ALB; CR; Education level; UA were adjusted. In the subgroup analysis stratified by GENDER, the model is not adjusted for GENDER. Weighted by: WTMEC2YR.

ALB = albumin, AST = aspartate transaminase, BMI = body mass index, CR = creatinine, PIR = income-to-poverty ratio, UA = uric acid.

**Figure 6.**
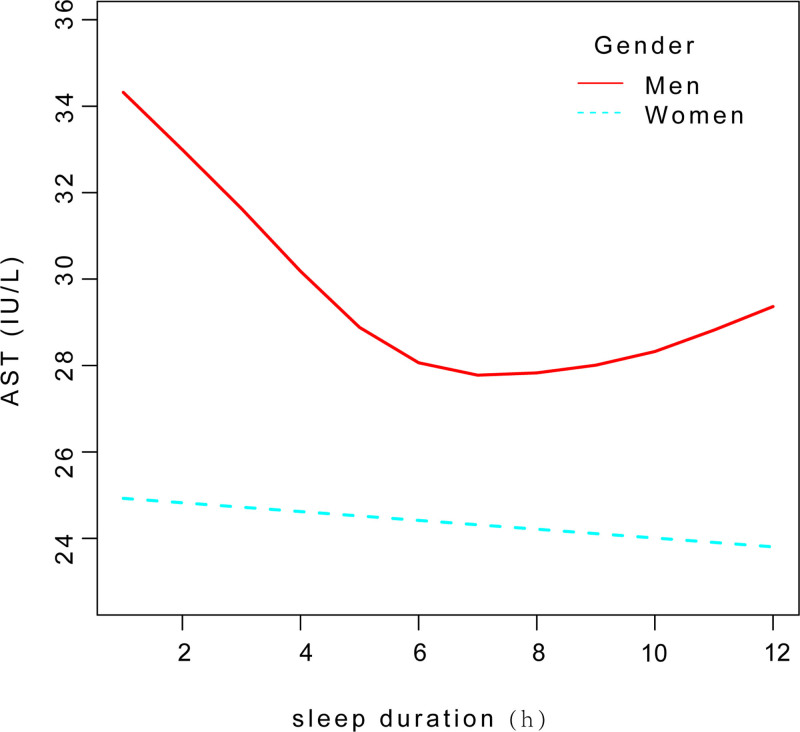
Smooth curve fit between sleep duration and AST level in subgroup analyses stratified by gender. AST = aspartate transaminase.

In subgroup analyses stratified by gender, our findings demonstrated a significant negative correlation in men. Using a two-segment linear regression model, in subgroup analyses stratified by gender, we found an U-shaped relationship between sleep duration and AST level with an inflection point of 5 hour and a significant log-likelihood ratio(*P *< .0001) in men(Fig. 6). A significant negative correlation was observed when sleep duration < 5 hours [−3.09 (−4.48, −1.70) < .0001]. Conversely, when sleep duration > 5 hours, a positive correlation was found between sleep duration and AST levels [0.09 (−0.28, 0.45) .6379](Table 9).

### 3.4. Association between sleep duration and TB

Table 10 shows the results of the multivariate regression analysis. In Model 2, sleep duration were highly positive correlated with TB level [0.07 (0.01, 0.13) .0134]. After adjusted for all covariates, this positive correlation change into insignificant in Model 3 [−0.02 (−0.09, 0.04)0.4386]. In the subgroup of stratified by gender, positive correlation was found between sleep duration and TB in men [0.10 (0.01, 0.19) .0233] and women[0.08 (0.01, 0.15) .0201] in Model 1.

**Table 10:** The association between sleep duration and TB

	Model 1 β (95% CI) *P* value	Model 2 β(95% CI) *P* value	Model 3 β (95% CI) *P* value
TB (μmol/L)	0.04 (−0.02, 0.10) .1504	0.07 (0.01, 0.13) .0134	−0.02 (−0.09, 0.04) .4386
Stratified by age			
AGE ≤ 40	0.03 (−0.07, 0.13) .5489	0.08 (−0.01, 0.18) .0763	−0.07 (−0.18, 0.05) .2575
AGE > 40	0.05 (−0.02, 0.12) .1440	0.05 (−0.02, 0.12) .1501	0.00 (−0.07, 0.07) .9573
Stratified by gender			
Men	0.10 (0.01, 0.19) .0233	0.08 (−0.01, 0.16) .0876	−0.06 (−0.16, 0.04) .2119
Women	0.08 (0.01, 0.15) .0201	0.07 (−0.00, 0.14) .0636	0.01 (−0.07, 0.09) .7934
Stratified by the tertile of BMI			
Low	0.06 (−0.05, 0.18) .2870	0.11 (−0.00, 0.22) .0546	0.02 (−0.11, 0.15) .7939
Middle	0.00 (−0.09, 0.10) .9794	−0.00 (−0.09, 0.09) .9572	−0.08 (−0.17, 0.02) .1276
High	−0.04 (−0.13, 0.05) .4365	−0.02 (−0.10, 0.07) .6551	−0.03 (−0.13, 0.06) .5065
Smoked at least 100 cigarettes			
Yes	0.04 (−0.03, 0.12) .2523	0.04 (−0.03, 0.12) .2538	−0.01 (−0.08, 0.07) .8881
No	−0.03 (−0.12, 0.07) .5952	0.00 (−0.09, 0.09) .9656	−0.05 (−0.15, 0.05) .3050

Model I: no covariates were adjusted. Model II: AGE, GENDER and Ethnicity were adjusted. Model III: AGE; GENDER; Ethnicity; PIR; Moderate activities; Smoked at least 100 cigarettes; BMI level; WAIST circumference; ALB; CR; Education level; UA were adjusted. In the subgroup analysis stratified by (age/gender/BMI/smoking status), the model is not adjusted for (age/gender/BMI/smoking status). Weighted by: WTMEC2YR.

ALB = albumin, AST = aspartate transaminase, BMI = body mass index, CR = creatinine, PIR = income-to-poverty ratio, UA = uric acid.

In the association between the quartile of sleep duration and TB, it revealed a statistically significant positive *P* value for trend in all Model (*P* for trend = 0.0093 in Model 2)(Table 11).

**Table 11:** The association between the tertile of sleep duration and TB

	Model 1 β (95% CI) *P* value	Model 2 β(95% CI) *P* value	Model 3 β (95% CI) *P* value
Tertile of sleep duration			
Low (1–5 h)	Reference	Reference	Reference
Middle (6–7 h)	0.56 (0.31, 0.80) < .0001	0.44 (0.20, 0.67) .0002	0.05 (−0.21, 0.30) .7194
High (8–12 h)	0.31 (0.05, 0.57) .0178	0.36 (0.11, 0.60) .0046	−0.08 (−0.35, 0.20) .5832
*P* for trend	0.09 (0.00, 0.17) .0470	0.11 (0.03, 0.19) .0093	−0.03 (−0.12, 0.06) .5181

Model I: no covariates were adjusted. Model II: AGE, GENDER and Ethnicity were adjusted. Model III: AGE; GENDER; Ethnicity; PIR; Moderate activities; Smoked at least 100 cigarettes; BMI level; WAIST circumference; ALB; CR; Education level; UA were adjusted. Weighted by: WTMEC2YR.

ALB = albumin, BMI = body mass index, CR = creatinine, PIR = income-to-poverty ratio, TB = total bilirubin, UA = uric acid.

Subgroup analyses were also conducted to investigate the association between sleep duration tertiles and TB levels (Table 12). Statistical significance was not found in those subgroups of TB level. Subgroup analysis showed that TB level of Other Hispanic group was more sensitive to sleep duration compared with another subgroup (*P* for trend = .0064).

**Table 12:** Subgroup analyses of the association between the tertile of sleep duration and TB

sleep duration	Low (1–5 h)	Middle (6–7 h)	High (8–12 h)	*P* for trend	*P* for interaction
Gender					.3520
Men	Reference	−0.25 (−0.65, 0.14) .2068	−0.29 (−0.71, 0.13) .1819	−0.09 (−0.23, 0.05) .1888	
Women	Reference	0.32 (−0.02, 0.65) .0649	0.11 (−0.24, 0.46) .5384	0.02 (−0.09, 0.14) .7179	
Age					.2569
≤ 40	Reference	−0.26 (−0.73, 0.21) .2820	−0.40 (−0.90, 0.10) .1156	−0.13 (−0.30, 0.03) .1132	
>40	Reference	0.24 (−0.06, 0.53) .1200	0.11 (−0.20, 0.42) .4941	0.03 (−0.08, 0.13) .5831	
Ethnicity(%)					.5900
Mexican American	Reference	0.02 (−0.61, 0.65) .9571	−0.01 (−0.66, 0.65) .9854	−0.00 (−0.22, 0.21) .9726	
Other Hispanic	Reference	−1.31 (−2.06, −0.55) .0007	−1.11 (−1.94, −0.29) .0083	−0.38 (−0.66, −0.11) .0064	
Non-Hispanic White	Reference	0.20 (−0.18, 0.57) .3008	0.02 (−0.38, 0.41) .9332	−0.01 (−0.14, 0.12) .8683	
Non-Hispanic Black	Reference	−0.01 (−0.62, 0.59) .9617	−0.15 (−0.83, 0.54) .6794	−0.04 (−0.26, 0.18) .7256	
Other race	Reference	−0.28 (−1.24, 0.68) .5705	−0.39 (−1.44, 0.66) .4705	−0.13 (−0.48, 0.22) .4686	
Moderate activities (%)					.0695
Yes	Reference	−0.07 (−0.46, 0.32) .7241	−0.31 (−0.73, 0.12) .1543	−0.11 (−0.25, 0.03) .1319	
No	Reference	0.12 (−0.22, 0.46) .5021	0.07 (−0.29, 0.42) .7129	0.02 (−0.10, 0.14) .7558	
Smoked at least 100 cigarettes					.4067
Yes	Reference	0.22 (−0.10, 0.54) .1811	0.01 (−0.33, 0.35) .9582	0.00 (−0.11, 0.11) .9903	
No	Reference	−0.19 (−0.60, 0.22) .3559	−0.26 (−0.69, 0.17) .2400	−0.08 (−0.23, 0.06) .2445	
Education level(%)					.6786
Less than high school	Reference	0.00 (−0.38, 0.39) .9827	−0.14 (−0.53, 0.26) .5004	−0.05 (−0.18, 0.09) .4985	
High school	Reference	0.11 (−0.36, 0.59) .6358	−0.28 (−0.78, 0.23) .2779	−0.10 (−0.27, 0.07) .2585	
More than high school	Reference	0.03 (−0.39, 0.46) .8746	−0.01 (−0.46, 0.44) .9664	−0.01 (−0.15, 0.14) .9313	
The tertile of BMI					.3715
Low	Reference	0.70 (0.11, 1.29) .0209	0.38 (−0.24, 0.99) .2296	0.08 (−0.12, 0.28) .4283	
Middle	Reference	−0.38 (−0.78, 0.02) .0612	−0.32 (−0.74, 0.10) .1414	−0.10 (−0.24, 0.04) .1777	
High	Reference	−0.09 (−0.46, 0.28) .6307	−0.29 (−0.69, 0.11) .1585	−0.10 (−0.23, 0.04) .1606	

AGE; GENDER; Ethnicity; PIR; Moderate activities; Smoked at least 100 cigarettes; BMI level; WAIST circumference; ALB; CR; Education level; UA were adjusted. In the subgroup analysis stratified by (AGE, Ethnicity, etc.), the model is not adjusted for (AGE, Ethnicity, etc.). Weighted by: WTMEC2YR.

ALB = albumin, BMI = body mass index, CR = creatinine, PIR = income-to-poverty ratio, TB = total bilirubin, UA = uric acid.

**Figure 7.**
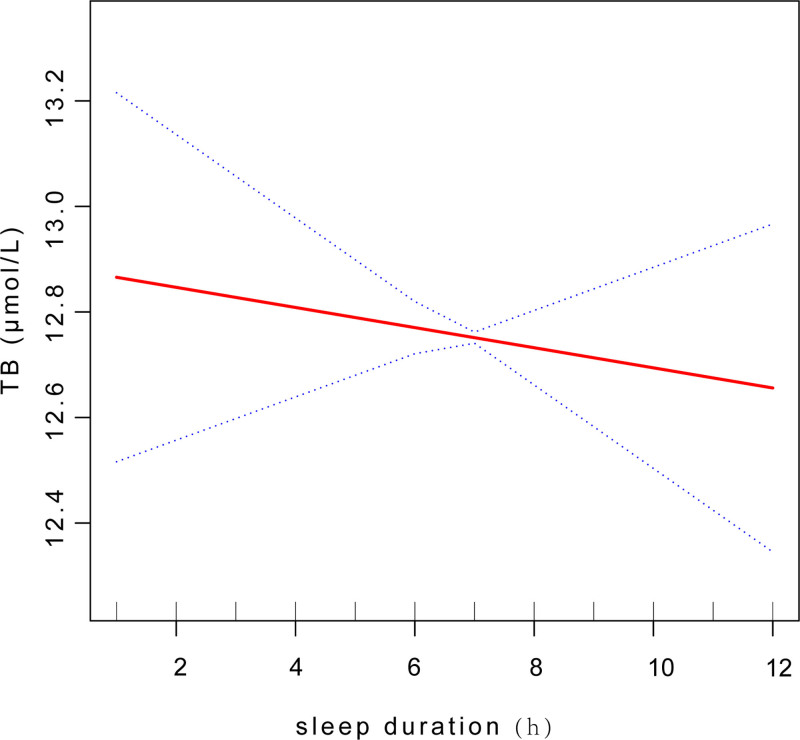
Smooth curve fit between sleep duration and TB level. TB = total bilirubin.

The nonlinear relationship was characterized by smooth curve fittings between sleep duration and TB level (Fig. 7).

## 4. Discussion

This study included a total of 17,491 participants, with a weighted average age of 45.58 years. Based on the NHANES database from 2005 to 2010, we present novel findings regarding the association between ALT, AST, and TB with sleep duration.

The results of this study revealed a significant inverse correlation between sleep duration and levels of ALT and AST, while no significant correlation was observed with TB. In a sleep deprivation trial, 64 healthy young male volunteers were kept awake for 76 to 80 hours. The blood tests conducted at the commencement and conclusion of continuous sleep deprivation revealed a mean elevation in AST levels by 170% and a mean increase in ALT levels by 58.5%.^[28]^ Another study indicated that both chronic sleep deprivation and acute sleep loss can induce oxidative stress in the liver and pancreas, leading to elevated levels of transaminases.^[29]^ These findings are consistent with our own observations. In a study investigating the impact of sleep on NAFLD and healthy populations, no significant correlation was observed between transaminases in the NAFLD group. However, in the healthy group, ALT levels exhibited a negative association with sleep duration, while AST concentrations showed a positive correlation with the total Pittsburgh sleep quality index score.^[30]^ These findings are consistent with our own observation. Nonetheless, the mechanism by which sleep duration is associated with liver function remains unknown.

Insomnia and sleep duration are influenced by various factors, including gender, health status, socio-demographic factors, environmental factors, genetic control and their combinations.^[31,32]^ We observed a higher proportion of males in the short sleep group compared to the long sleep group and a significantly higher proportion of females in the long sleep group. Individuals with higher levels of education and income exhibit a greater prevalence of long sleep duration, while those with lower educational attainment demonstrate a higher incidence of short sleep duration. Additionally, there was an association between increased BMI and waist circumference with shorter sleep duration. The percentage of smokers was greater in the population experiencing short-sleep duration; conversely, nonsmokers constituted a larger proportion within the long-sleep group. Of the racial differences, Non-Hispanic Black and Other Hispanic had significantly higher proportions of the short-sleep group, and Non-Hispanic White and Mexican American had higher proportions of the long-sleep group.

Interestingly, the connection between sleep duration and mortality is similar to the association between sleep and inflammation.^[12]^ Strong evidence shows that sleep disorders can have an adverse impact on the risk of inflammatory diseases and lead to an increase in all-cause mortality.^[33]^ Some research reports on the results of a meta-analysis of sleep time and mortality found a U-shaped association. Among them, long sleepers (>8 hours per night) have a 30% higher risk, while short sleepers (<7 hours per night) have an 8% higher risk of death than those who sleep 7 to 12 hours per night.^[12]^ At present, the mechanism of the relationship between sleep disorders and inflammation remains unclear. Sleep affects 2 major effector systems, namely the hypothalamic-pituitary-adrenal axis and the sympathetic nervous system, which together shift the basal gene expression profile towards an increased pro-inflammatory bias.^[34,35]^ Activation of β-adrenergic signal transduction induces an increase in nuclear factor(NF)-κB, inflammatory gene expression, production of pro-inflammatory cytokines, and systemic inflammatory markers.^[34]^ Studies have shown that one of the reasons why cells produce IL-6 and tumor necrosis factor (TNF) is the activation of toll-like receptor (TLR) activity. Partial lack of sleep at night can induce an increase in TLR-4-stimulated inflammatory cytokine production.^[36]^ Given that normal nighttime sleep is associated with a decrease in sympathetic outflow,^[37]^ activation of the sympathetic effector pathway is a biologically plausible mechanism that can explain the association between sleep disorders, short sleep duration, and increased inflammatory markers.^[38]^ In addition, a prospective study showed that short sleep duration is associated with inflammatory markers such as C-reactive protein (CRP)^[39]^ and interleukin 6 (IL-6).^[40]^ A meta-analysis found that compared with a sleep duration of 7-8 hours, a sleep duration of > 8 hours is associated with an increase in CRP and IL-6.^[38]^ Moreover, a certain study has indicated that when subjects sleep only 4 hours per night for twelve consecutive days and are in a prolonged state of sleep restriction, the level of IL-6 will increase.^[41]^ We believe that the U-shaped relationship between sleep duration and inflammatory factor levels is not a coincidence. There may also be an optimal sleep duration for liver function recovery.

There are many factors that affect liver function. Given that a large number of studies have shown the impact of sleep on inflammatory factors, the influence of inflammation on liver function has attracted sufficient attention. For example, the occurrence of NAFLD is characterized by inflammation, and hepatic steatosis and fibrosis are significantly affected by the migration of circulating inflammatory cells and the overexpression of inflammatory mediators.^[42]^ Clinical parameters of plasma cytokines and inflammation can be used as a new strategy for monitoring the progression of NAFLD.^[43]^ Studies have shown that elevated serum hs-CRP levels are associated with an increased risk of Metabolic-Associated Fatty Liver Disease in obese Chinese patients and are positively correlated with the severity of hepatic steatosis and fibrosis.^[44]^ Moreover, CRP levels can predict the survival rate of patients with cirrhosis.^[45]^ When the liver is subjected to ischemia/reperfusion injury, a large amount of IL-6 in the microenvironment will aggravate liver injury, such as intrahepatic tissue damage and inflammation.^[46]^ If repeated or chronic liver IL-6 inflammatory injury is elevated, it will trigger a series of liver diseases. Many studies have emphasized that long-term exposure to IL-6 will impair liver lipid metabolism.^[47–49]^ In a humanized liver mouse model, the inflammatory effect of the IL-6/Glycoprotein 130 pathway promotes liver lipid accumulation, indicating the therapeutic potential of antagonizing Glycoprotein 130 signal transduction in the treatment of hepatic steatosis.^[50]^ In a mouse model of nonalcoholic steatohepatitis (NASH), knockout of IL-6 or IL-6R also reduces the signs of inflammation during NASH progression.^[51]^ In addition, a large number of studies have shown that inflammation and inflammasome activation are related to the development of liver diseases.^[52–54]^ Insufficient sleep duration and poor sleep quality may contribute to insulin resistance and subclinical inflammation,^[55]^ which can subsequently lead to alterations in transaminase levels due to inflammatory cytokines such as TNF-α and IL-6.^[56]^ Hence, we hypothesize that sleep duration may potentially influence liver function through inflammation-related pathways.

In the relationship between sleep duration and ALT, we examined the specific situations of the age, gender, and BMI subgroups. The results of Model 2 showed that among individuals aged ≤ 40 years [−0.64 (−0.94, −0.34) < .0001], men [−0.75 (−1.08, −0.43) < .0001], low-BMI group [−0.34 (−0.58, −0.11) .0046], and nonsmokers [−0.48 (−0.75, −0.21) .0006], all exhibited significant negative correlations. The same situation occurred in the subgroup analysis of the relationship between sleep duration and AST. In Model 2, we found that there were also significant negative correlations among the population with age ≤ 40 years, the male population, and the nonsmoking population. Their statistical results were [−0.30 (−0.54, −0.06) .0162], [−0.34 (−0.58, −0.10) .0047], and [−0.26 (−0.50, −0.03) .0280] respectively. Based on the results of the subgroup analysis, We suggest a potential negative association between sleep duration and liver function recovery in younger (≤40 years), males, and nonsmokers groups. Furthermore, The results of the trend test suggested that both ALT and AST levels showed a positive trend for sleep duration, indicating that this negative correlation was more significant as sleep duration increased; however this positive trend varied across subgroups. Specifically, ALT levels were more sensitive to changes in sleep duration among Non-Hispanic White individuals while AST levels showed greater sensitivity among Non-Hispanic Black individuals as well as those who did not engage in moderate exercise or smoker (>100 cigarettes) or high school education population.

Both short and long sleep durations have been associated with a range of adverse health outcomes. We hypothesize that there must be an optimal sleep duration for liver function recovery and we endeavor to determine this optimal sleep duration for different populations through this study. Therefore, we observed significant inflection points through smoothed curve fitting and threshold effect analysis. Our findings regarding the relationship between sleep duration and ALT levels showed that in individuals aged ≤ 40 years, ALT levels were lowest at 8 hours of sleep, while no significant inflection point was found in those aged > 40 years. It is evident that among young individuals (aged ≤ 40 years) with short sleep duration (<8 hours), adequate sleep contributes to lower ALT levels and promotes liver function recovery. In middle-BMI and high-BMI groups, ALT levels were lowest at 5 and 8 hours respectively, suggesting that the optimal duration for liver function recovery may be longer in populations with higher BMI compared to lower BMI. AST levels were lowest when individuals slept for 5 hours. In the subgroup of gender, males exhibited the lowest AST levels when sleeping for 5 hours. These findings suggest that shorter sleep durations (<5 hours) are protective factors for liver function. From the perspective of inflammation, we have found evidence of some subgroup differences to explain why there is no optimal sleep duration for liver function recovery in some populations. In adolescents, a healthy immune status is associated with moderate sleep duration.^[57]^ However, in people with excessive sleep duration, circulating inflammatory markers such as interleukins, CRP, and TNF-α will increase.^[57]^ IL-6 is one of the major Senescence-Associated Secretory Phenotype factors and also belongs to pro-inflammatory cytokines.^[58]^ In the serum of healthy adults, IL-6 is usually lower than 2 pg/mL or undetectable, and the serum IL-6 content gradually increases with age.^[59,60]^ In the aged liver, IL-6 can be expressed and secreted by all senescent liver cells, suggesting its multifaceted roles in the development of age-related diseases.^[61]^ In addition, a large number of studies support the potential correlation between IL-6 levels and aging and chronic morbidity.^[58,62]^ Moreover, the activation of IL-6 effector signals is positively correlated with age-related lipid metabolism disorders, hepatitis, and fibrosis.^[63,64]^ This may be the reason for the different results in age subgroups. In addition, there are also gender differences in the expression of inflammatory factors under the influence of sleep duration. Compared with men, women may be more vulnerable to sleep disorders, with greater increases in CRP and IL-6,^[65,66]^ greater increases in inflammatory cytokines produced by TLR-4-stimulated monocytes, and greater increases in NF-κB.^[67,68]^ Liu et al^[69]^ found that poor sleep quality is associated with elevated plasma hs-CRP levels in women but not in men among the adult population in the United States. Jackowska et al^[70]^ found that long sleep duration was associated with increased CRP only in men, and Miller et al^[71]^ found that short sleep duration was linked to high hs-CRP levels only in women. This suggests that there may be gender differences in the impact of sleep on the inflammatory response, but the specific reasons for these differences need to be further explored.

In the relationship between sleep duration and TB, a significant positive correlation was observed in the Model 2; After adjusting for all covariates, the relationship became non-significant in Model 3. This observation may be attributed to the multitude of influential factors affecting TB. In addition to being associated with liver function, TB is also related to various factors such as the biliary system and hemolysis.

This study has certain implications. Firstly, our findings can provide supportive evidence for clinical work. The results of this study provide evidence that healthy sleep is important for liver function recovery. And we found that sleep is more important for liver function recovery in older individuals (>40 years), male population and nonsmokers. Secondly, Our findings indicate that individuals in the ≤ 40 age group exhibited the lowest levels of ALT when their sleep duration was 8 hours, while among males, AST levels were found to be lowest with a sleep duration of 5 hours. We should adjust the sleep duration to ensure the optimal sleep duration. Different gpopulations of people should select an appropriate optimal sleep duration, which is conducive to the recovery of liver function.

The study has certain limitations. Firstly, the cross-sectional study design prevents establishing a causal relationship between ALT, AST, and TB levels and sleep duration. Conducting future longitudinal studies could enhance the reliability of these findings. Secondly, self-reported information was used to assess sleep duration and sleep time. However, it is important to note that self-reported or perceived sleep duration may differ from objective assessments obtained through activity recorders,^[72,73]^ potentially introducing recall bias and information bias.^[74]^ Nevertheless, sleep diaries, actigraphy, and polysomnography from some large population-based studies and small-scale surveys have demonstrated a high degree of correlation with subjective estimates of sleep duration.^[75,76]^ Furthermore, despite adjusting for several relevant confounders, we are still unable to fully eliminate the potential impact of other confounding factors. Despite these limitations, our study possesses several strengths. By utilizing a nationally representative sample, our research encompasses a diverse population of adults in the United States in terms of ethnicity and gender. Additionally, the inclusion of a large sample size enabled us to conduct subgroup analyses.

## 5. Conclusion

The present study unveiled a significant inverse correlation between sleep duration and levels of ALT and AST, while no significant association was observed with TB levels. Furthermore, variations in the optimal sleep duration for liver function recovery were identified across diverse populations, thereby offering valuable healthcare recommendations to the general public.

## Author contributions

**Conceptualization:** Tiantian Chen.

**Data curation:** Lishuai Yao, Tiantian Chen.

**Formal analysis:** Lishuai Yao, Tiantian Chen.

**Software:** Lishuai Yao, Tiantian Chen.

**Supervision:** Tiantian Chen.

**Writing – original draft:** Lishuai Yao, Tiantian Chen.

**Writing – review & editing:** Tiantian Chen.
